# Bullet Extracted through Urethra

**Published:** 1915-03

**Authors:** 


**Bullet Extracted Through Urethra.** Zondek, Berliner Klin. Woch., Dec. 7, 1914, reports a case of a soldier struck by a narrow, pointed Russian machine-gun missile, close to the urinary meatus. The bullet could be felt in the penis but five days later it escaped into the bladder, being located by X-Rays in front of the symphysis. A specially devised bullet-catcher was used for its removal.

